# Receipt of COVID-19 vaccine in preterm-born children aged 3-7 in China

**DOI:** 10.3389/fpubh.2023.1191941

**Published:** 2023-07-20

**Authors:** Dan Wang, Li Li, Xiaofeng Ma, Yunfeng Lin, Liping Chen, Arigonggaowa, Xianglian Peng, Jingyun Shi, Jie Yang, Rong Ju, Zhankui Li, Jingke Cao, Changgen Liu, Zhichun Feng, Qiuping Li

**Affiliations:** ^1^Newborn Intensive Care Unit, Faculty of Pediatrics, The Seventh Medical Center of Chinese People's Liberation Army (CPLA) General Hospital, Beijing, China; ^2^The Second School of Clinical Medicine, Southern Medical University, Guangzhou, China; ^3^State Key Laboratory of Organ Failure Research, Department of Biostatistics, Guangdong Provincial Key Laboratory of Tropical Disease Research, School of Public Health, Southern Medical University, Guangzhou, China; ^4^Department of Neonatology, Fujian Children's Hospital (Fujian Branch of Shanghai Children's Medical Center), College of Clinical Medicine for Obstetrics & Gynecology and Pediatrics, Fujian Medical University, Fuzhou, China; ^5^Department of Neonatology, Fujian Maternity and Child Health Hospital, College of Clinical Medicine for Obstetrics & Gynecology and Pediatrics, Fujian Medical University, Fuzhou, China; ^6^Fujian Key Laboratory of Women and Children's Critical Diseases Research (Fujian Maternity and Child Health Hospital), Fuzhou, China; ^7^Newborn Intensive Care Unit, Jiangxi Provincial Children's Hospital, Nanchang, China; ^8^Department of Neonatology, Inner Mongolia Maternal and Child Health Hospital, Hohhot, China; ^9^Department of Neonatology, Hunan Provincial Maternal and Child Health Care Hospital, Changsha, China; ^10^Department of Neonatology, Gansu Provincial Maternal and Child Hospital, Lanzhou, China; ^11^Department of Neonatology, Nanfang Hospital, Southern Medical University, Guangzhou, China; ^12^Department of Neonatology, Chengdu Women's and Children's Central Hospital, School of Medicine, University of Electronic Science and Technology of China, Chengdu, China; ^13^Department of Neonatology, Northwest Women's and Children's Hospital (Maternal and Child Health Care Hospital of Shaanxi Province), Xi'an, China

**Keywords:** pediatrics, preterm-born children, COVID-19, inactivated vaccine, vaccination

## Abstract

**Objectives:**

To determine the COVID-19 vaccination rate in preterm-born children aged 3–7 in China and influential factors, identify vaccination-related adverse reactions, and clarify reasons behind parental refusal of vaccination to their children.

**Methods:**

This cross-sectional study was conducted in parents of preterm-born children aged 3–7 in different regions of China through WeChat.

**Results:**

Of 1,924 Chinese preterm-born children aged 3–7 included in this study, 1,552 (80.7%) had been vaccinated against COVID-19, with a higher vaccination rate in eastern China. Children older than 4 years, kids in kindergartens and primary schools, children living in eastern or western China, and children whose fathers had received at least one dose of a COVID-19 vaccine tended to be vaccinated against COVID-19 after adjusting for other covariates. Conversely, children living in urban areas, children whose annual family income was < 50,000 CNY or more than 300,000 CNY, premature children who underwent hospital transfers, and children with underlying diseases were less likely to get vaccinated. Adverse reactions occurred in 59/1,552 (3.8%) vaccinated children. Parents of 118/372 (31.7%) children expressed their concerns over adverse reactions to COVID-19 vaccination. Other reasons like the absence of information about the place where they could get vaccination were mentioned as well.

**Conclusions:**

COVID-19 vaccination-related adverse reactions rarely occurred and most of them were mild among preterm-born children aged 3–7. Higher vaccination coverage can be achieved as parents are provided with more scientific data about benefits of vaccination, vaccination contraindications and precautions, and more information about vaccination sites.

## 1. Introduction

The outbreak of COVID-19 has posed a serious threat to the health and life of children. By June 1, 2022, about 21 million children under 10 had contracted COVID-19 infection in more than 103 countries and regions. Meanwhile, ~8,000 children under 10 died from the COVID-19 pandemic in about 91 countries and regions (1). Currently, Omicron variants are still circulating worldwide and transmissibility of those variants is higher than that of other variants (2). Infections from the Omicron variants seem to require more health-care services. For instance, the hospitalization rate of children under 5 during the epidemic peak of Omicron variants was about four times higher than that during the Delta variant epidemic peak (3). Compared with the direct impact of COVID-19 on child and youth mortality, the indirect impact of the disruptions to care-seeking and preventative interventions such as vaccination has become more substantial in the world (4).

Preterm-born children are defined as children born < 37 weeks' gestation, with varying degrees of immaturity in body organs and physiological functions, especially those with a gestational age < 32 weeks who may have some complications or stay in the hospital for a prolonged period after birth. In China, 1.2 million premature babies born every year, and the incidence of premature birth is about 7% (5). There was a study showing that preterm-born children were likely to be vulnerable to severe acute respiratory syndrome coronavirus 2 (SARS-CoV-2) infection and had a high risk of severe COVID-19, and these children needed priority for vaccination against SARS-CoV-2 (6). A certain number of preterm-born children are likely to be vulnerable to SARS-CoV-2 infection and therefore these children should be protected well during the COVID-19 pandemic. However, there is no specific medicine for children under 12 with COVID-19 at present. Fortunately, clinical studies have proved that inactivated COVID-19 vaccines, such as CoronaVac and BBIBP-CorV are safe for children aged 3–17 years (7, 8). In addition, real-world research has also verified that inactivated vaccines can be used for children aged 3 and older by providing protection against infection, hospitalization, and progression to critical illness caused by Omicron variants (9–11). Vaccination of children is believed to help achieve herd immunity, avoid severe COVID-19, and prevent the emergence of new COVID-19 variants (12). In China, children aged 3 and older can receive COVID-19 vaccination since 2021 (13, 14).

COVID-19 vaccination is contraindicated in three groups of individuals: (1) individuals who are allergic to an ingredient contained in the novel coronavirus vaccine; (2) individuals with a previous history of severe allergy to vaccines such as acute allergic reactions, angioedema, and dyspnea; and (3) individuals who have ever had adverse reactions to COVID-19 vaccines (excluding low-grade fever, local general reactions such as swelling and pain) (15). According to the expert consensus on COVID-19 vaccination and guideline for preterm-born children vaccination, preterm-born children can accept COVID-19 vaccines (5, 15). Since the beginning of December 2022, China has gradually liberalized its COVID-19 management policies. Since preterm-born children are at elevated risk of SARS-CoV-2 infection, it is of great importance to maintain a high herb immunity among these children through vaccination. To increase the vaccination rate and thereafter minimize the adverse impact of COVID-19 on preterm-born children, it is imperative to gain a better understanding about the COVID-19 vaccination coverage and potential influential factors, identify vaccination-related adverse reactions in these children, and clarify reasons behind parental refusal to vaccinate their children. This study aims to estimate the COVID-19 vaccination rate in preterm-born children aged 3–7 in different regions of China, explore influential factors of the vaccination status, identify adverse reactions pertaining to COVID-19 vaccination in these children, and clarify reasons of their parents' refusal to have their children vaccinated.

## 2. Methods

### 2.1. Study design

In September 2022, we conducted a survey among parents of preterm-born children aged 3–7 in China. Inclusion criteria were: (1) preterm-born children who were born < 37 weeks' gestation; (2) children who were born in Chinese mainland. Preterm-born children whose information was incomplete were excluded. A standardized questionnaire form was designed after repeated discussions and revisions by hospital senior neonatal experts. The questionnaire was developed by “Wenjuanxing (Questionnaire Star),” an online survey platform, and distributed in WeChat groups including the groups of neonatology department directors from the west, center and east of China. Then, the questionnaires were forwarded to parents of preterm-born children aged 3–7. This study was approved by the research ethics board of our hospital in north of China. This survey was completed freely.

### 2.2. Sample size calculation

We estimated the minimum sample size using the following equation:


$$\begin{array}{l} n=\frac{z_{\alpha /2}^{2}\pi \left(1-\pi \right)}{\delta^{2}}\times \frac{1}{r}\times deff \end{array}$$

where *n* is the sample size; *z*_α/2_ is the statistics corresponding to the confidence level (i.e., 95%); π is the expected vaccination rate (i.e., 50% which will produce the largest sample size); δ is precision (i.e., 5%); *r* is the expected response rate (i.e., 80%); *deff* is design effect (i.e., 2). The minimum sample size required was 961.

### 2.3. Data collection

Data of demographics, health-related information, delay in the first inoculation, and the COVID-19 vaccination status of the parents were collected. Demographic data included age, sex, education level, one child or not, ethnic group, parents' education level, place of residence (i.e., west, center, and east. The division of western, central, and eastern regions of Chinese mainland was based on the level of economic development and geographical location) (16), annual family income, and medical insurance. Health-related information included maternal pregnancy-related information such as the way of conception and maternal age; the previous health status such as gestational age, birth weight, whether being premature children who underwent hospital transfers or admitted to a neonatology unit at birth, severity of bronchopulmonary dysplasia at first hospital discharge, whether having congenital heart disease or brain damage at first hospital discharge; and the recent health status such as the existing diseases, histories of recurrent infections and milk or food allergy in the past 6 months, current weight, and any history of hospitalization in the past year. For vaccinated children, we also obtained information on whether they were fully vaccinated, the reasons for getting vaccinated, and the vaccination-related adverse reactions. For unvaccinated children, the reasons for refusing to get the vaccination and parents' willingness to have their children vaccinated if their children had no evidence of COVID-19 vaccine contraindications.

### 2.4. Statistical analysis

According to their COVID-19 vaccination status, the included preterm-born children were classified into two groups: vaccinated vs. unvaccinated. We summarized categorical and ordinal variables by counts and the corresponding percentages. Meanwhile, continuous variables were expressed as the mean and standard deviation. Modified Poisson regression models were applied to determine the influential factors of COVID-19 vaccination in preterm-born children aged 3–7 in China. First, we conducted univariate analysis with candidate independent variables including the demographic data, health-related information, and the status of COVID-19 vaccination of the parents. Factors with *P*-values < 0.1 in univariate analysis were subjected to multivariate analysis. We estimated the prevalence ratio (PR) of COVID-19 vaccination for each category to compare it with the reference group, and the corresponding 95% confidence interval (CI) of PR was provided as well. All analyses were conducted with R software version 4.1.3 (R Foundation for Statistical Computing).

## 3. Results

The response rate was 80.2% (1,924/2,400). Of the 1,924 Chinese preterm-born children aged 3–7 included in this study, 1,552 (80.7%, 95% CI: 78.8–82.4%) had been vaccinated against COVID-19 and 1,390 (72.2%, 95% CI: 70.2–74.2%) had received two shots of the vaccines. In China, the COVID-19 vaccination coverage varied across regions. The vaccination rate in the included preterm-born children was 75.2% (95% CI: 71.9–78.3%), 76.9% (95% CI: 73.2–80.3%), and 88.7% (95% CI: 86.2–90.8%) in the west, center, and east of China, respectively. The percentages of children living in urban areas were 86.4% (593/686), 63.9% (338/529), and 68.0% (482/709) in the western, central, and eastern regions of China, respectively. The vaccination rates standardized by the urban percentage (standard population: all subjects in this study) were 74.7% (95% CI: 67.8–81.6%), 75.2% (95% CI: 67.7–82.7%), and 88.8% (95% CI: 81.8–95.8%) in the west, center, and east of China, respectively. It was found that the vaccinated children were on average older than the unvaccinated (5.13 vs. 4.06 years). 179/1,924 (9.3%) participated children suffered from at least one disease currently. Over 98% of the fathers and the mothers had received COVID-19 vaccines (Table 1).

**Table 1:** Demographic data, health-related information, delay in the first dose of vaccination, and parents' COVID-19 vaccination status of preterm-born children aged 3–7 years.

**Variables**	**All**	**Vaccinated**	**Unvaccinated**	**PR (95% CI)^a^**	***P* ^b^**
**Demographics**					
**Age group, years**					< 0.001
3–4	429/1,924 (22.3)	212/1,552 (13.7)	217/372 (58.3)	Reference	
4–5	539/1,924 (28.0)	443/1,552 (28.5)	96/372 (25.8)	1.66 (1.50–1.84)	< 0.001
5–6	578/1,924 (30.0)	542/1,552 (34.9)	36/372 (9.7)	1.90 (1.72–2.09)	< 0.001
6–7	378/1,924 (19.6)	355/1,552 (22.9)	23/372 (6.2)	1.90 (1.72–2.10)	< 0.001
**Sex**					
Male	1,070/1,924 (55.6)	868/1,552 (55.9)	202/372 (54.3)	1.01 (0.97–1.06)	0.572
Female	854/1,924 (44.4)	684/1,552 (44.1)	170/372 (45.7)	Reference	
**Education level**					< 0.001
Pre-kindergarten	147/1,924 (7.6)	32/1,552 (2.1)	115/372 (30.9)	Reference	
Kindergarten	1,427/1,924 (74.2)	1,176/1,552 (75.8)	251/372 (67.5)	3.79 (2.78–5.15)	< 0.001
Primary school	350/1,924 (18.2)	344/1,552 (22.2)	6/372 (1.6)	4.52 (3.32–6.14)	< 0.001
**One child**					
Yes	756/1,924 (39.3)	607/1,552 (39.1)	149/372 (40.1)	0.99 (0.95–1.04)	0.739
No	1,168/1,924 (60.7)	945/1,552 (60.9)	223/372 (59.9)	Reference	
**Han Chinese**					
Yes	1,835/1,924 (95.4)	1,481/1,552 (95.4)	354/372 (95.2)	1.01 (0.91–1.13)	0.831
No	89/1,924 (4.6)	71/1,552 (4.6)	18/372 (4.8)	Reference	
**Father's education level**					0.136
Junior high school or below	370/1,924 (19.2)	302/1,552 (19.5)	68/372 (18.3)	Reference	
High school	541/1,924 (28.1)	451/1,552 (29.1)	90/372 (24.2)	1.02 (0.96–1.09)	0.499
University	911/1,924 (47.3)	722/1,552 (46.5)	189/372 (50.8)	0.97 (0.92–1.03)	0.325
Postgraduate or above	102/1,924 (5.3)	77/1,552 (5.0)	25/372 (6.7)	0.92 (0.82–1.04)	0.205
**Mother's education level**					0.589
Junior high school or below	419/1,924 (21.8)	338/1,552 (21.8)	81/372 (21.8)	Reference	
High school	532/1,924 (27.7)	439/1,552 (28.3)	93/372 (25.0)	1.02 (0.96–1.09)	0.467
University	902/1,924 (46.9)	719/1,552 (46.3)	183/372 (49.2)	0.99 (0.93–1.05)	0.683
Postgraduate or above	71/1,924 (3.7)	56/1,552 (3.6)	15/372 (4.0)	0.98 (0.86–1.11)	0.733
**Region**					< 0.001
Center of China	529/1,924 (27.5)	407/1,552 (26.2)	122/372 (32.8)	Reference	
East of China	709/1,924 (36.9)	629/1,552 (40.5)	80/372 (21.5)	1.15 (1.09–1.22)	< 0.001
West of China	686/1,924 (35.7)	516/1,552 (33.2)	170/372 (45.7)	0.98 (0.92–1.04)	0.485
**Living in urban areas**					
Yes	1,413/1,924 (73.4)	1,116/1,552 (71.9)	297/372 (79.8)	0.93 (0.89–0.97)	0.001
No	511/1,924 (26.6)	436/1,552 (28.1)	75/372 (20.2)	Reference	
**Annual family income, CNY** ^c^					0.061
≤ 50,000	231/1,924 (12.0)	169/1,552 (10.9)	62/372 (16.7)	0.90 (0.83–0.98)	0.014
50,000–150,000	988/1,924 (51.4)	803/1,552 (51.7)	185/372 (49.7)	Reference	
150,000–300,000	545/1,924 (28.3)	448/1,552 (28.9)	97/372 (26.1)	1.01 (0.96–1.06)	0.652
>300,000	160/1,924 (8.3)	132/1,552 (8.5)	28/372 (7.5)	1.02 (0.94–1.10)	0.705
**Medical insurance**					
The option to pay off medical bills					0.605
Out–of–pocket	143/1,924 (7.4)	116/1,552 (7.5)	27/372 (7.3)	Reference	
Rural cooperative medical insurance	676/1,924 (35.1)	553/1,552 (35.6)	123/372 (33.1)	1.01 (0.92–1.10)	0.849
Urban resident basic medical scheme	1,105/1,924 (57.4)	883/1,552 (56.9)	222/372 (59.7)	0.99 (0.91–1.07)	0.727
**Having commercial insurance**					
Yes	517/1,924 (26.9)	417/1,552 (26.9)	100/372 (26.9)	1.00 (0.95–1.05)	0.996
No	1,407/1,924 (73.1)	1,135/1,552 (73.1)	272/372 (73.1)	Reference	
**Maternal pregnancy related information**					
**Natural conception**					
Yes	1,670/1,924 (86.8)	1,368/1,552 (88.1)	302/372 (81.2)	1.13 (1.04–1.22)	0.002
No	254/1,924 (13.2)	184/1,552 (11.9)	70/372 (18.8)	Reference	
**Maternal age, years**					0.212
25–29	752/1,924 (39.1)	614/1,552 (39.6)	138/372 (37.1)	Reference	
≤ 25	196/1,924 (10.2)	154/1,552 (9.9)	42/372 (11.3)	0.96 (0.89–1.04)	0.350
30–35	669/1,924 (34.8)	527/1,552 (34.0)	142/372 (38.2)	0.96 (0.92–1.02)	0.176
≥35	307/1,924 (16.0)	257/1,552 (16.6)	50/372 (13.4)	1.03 (0.97–1.09)	0.414
**Previous health status**					
**Gestational age, weeks**					
< 32	380/1,924 (19.8)	289/1,552 (18.6)	91/372 (24.5)	0.93 (0.87–0.99)	0.019
≥32	1,544/1,924 (80.2)	1,263/1,552 (81.4)	281/372 (75.5)	Reference	
Birth weight, kg	2.09 ± 0.58	2.10 ± 0.57	2.04 ± 0.62	1.03 (0.99–1.07)	0.120
**Premature children who underwent hospital transfers**					
Yes	257/1,924 (13.4)	191/1,552 (12.3)	66/372 (17.7)	0.91 (0.84–0.98)	0.015
No	1,667/1,924 (86.6)	1,361/1,552 (87.7)	306/372 (82.3)	Reference	
**Admitted to a neonatology unit at birth**					
Yes	1,798/1,924 (93.5)	1,457/1,552 (93.9)	341/372 (91.7)	1.07 (0.97–1.19)	0.167
No	126/1,924 (6.5)	95/1,552 (6.1)	31/372 (8.3)	Reference	
**Severity of BPD at first hospital discharge**					0.276
No	1,664/1,924 (86.5)	1,353/1,552 (87.2)	311/372 (83.6)	Reference	
Mild	162/1,924 (8.4)	126/1,552 (8.1)	36/372 (9.7)	0.96 (0.88–1.04)	0.308
Moderate	58/1,924 (3.0)	46/1,552 (3.0)	12/372 (3.2)	0.98 (0.85–1.11)	0.715
Severe	40/1,924 (2.1)	27/1,552 (1.7)	13/372 (3.5)	0.83 (0.67–1.03)	0.092
**Congenital heart disease at first hospital discharge**					
Yes	541/1,924 (28.1)	431/1,552 (27.8)	110/372 (29.6)	0.98 (0.94–1.03)	0.495
No	1,383/1,924 (71.9)	1,121/1,552 (72.2)	262/372 (70.4)	Reference	
**Patent ductus arteriosus**					
Yes	278/1,924 (14.4)	231/1,552 (14.9)	47/372 (12.6)	1.04 (0.98–1.10)	0.242
No	1,646/1,924 (85.6)	1,321/1,552 (85.1)	325/372 (87.4)	Reference	
**Atrial septal defect**					
Yes	114/1,924 (5.9)	77/1,552 (5.0)	37/372 (9.9)	0.83 (0.73–0.94)	0.004
No	1,810/1,924 (94.1)	1,475/1,552 (95.0)	335/372 (90.1)	Reference	
**Brain damage at first hospital discharge**					
Yes	336/1,924 (17.5)	310/1,552 (20.0)	26/372 (7.0)	1.18 (1.13–1.23)	< 0.001
No	1,588/1,924 (82.5)	1,242/1,552 (80.0)	346/372 (93.0)	Reference	
**Delay in the first inoculation**					
Yes	1,231/1,924 (64.0)	974/1,552 (62.8)	257/372 (69.1)	0.95 (0.91–0.99)	0.019
No	693/1,924 (36.0)	578/1,552 (37.2)	115/372 (30.9)	Reference	
**Recent health status**					
**Currently suffering from underlying diseases** ^d^					
Yes	179/1,924 (9.3)	127/1,552 (8.2)	52/372 (14.0)	0.87 (0.79–0.96)	0.004
No	1,745/1,924 (90.7)	1,425/1,552 (91.8)	320/372 (86.0)	Reference	
**Recurrent infections**					
Yes	96/1,924 (5.0)	71/1,552(4.6)	25/372(6.7)	0.91(0.81–1.03)	0.139
No	1,828/1,924 (95.0)	1,481/1,552(95.4)	347/372(93.3)	Reference	
**Milk or food allergy in the past 6 months**					
Yes	134/1,924 (7.0)	107/1,552 (6.9)	27/372 (7.3)	0.99 (0.91–1.08)	0.808
No	1,790/1,924 (93.0)	1,445/1,552 (93.1)	345/372 (92.7)	Reference	
**Current weight, kg**	18.35 ± 4.17	18.83 ± 3.96	16.38 ± 4.45	1.03 (1.02–1.03)	< 0.001
**Being hospitalized in the past year**					
Yes	461/1,924 (24.0)	388/1,552 (25.0)	73/372 (19.6)	1.06 (1.01–1.11)	0.020
No	1,463/1,924 (76.0)	1,164/1,552 (75.0)	299/372 (80.4)	Reference	
**COVID-19 vaccination status of parents**					
**Father's vaccination status**					
Yes	1,908/1,924 (99.2)	1,545/1,552 (99.5)	363/372 (97.6)	1.85 (1.06–3.23)	0.030
No	16/1,924 (0.8)	7/1,552 (0.5)	9/372 (2.4)	Reference	
**Mather's vaccination status**					
Yes	1,890/1,924 (98.2)	1,533/1,552 (98.8)	357/372 (96.0)	1.45 (1.08–1.96)	0.015
No	34/1,924 (1.8)	19/1,552 (1.2)	15/372 (4.0)	Reference	

Categorical variables are expressed as counts and the corresponding percentages, while continuous variables are summarized as the mean ± standard deviation.

COVID-19, coronavirus disease 2019; 95% CI, 95% confidence interval; PR, prevalence ratio; CNY, Chinese yuan renminbi; BPD, bronchopulmonary dysplasia.

^a^Prevalence ratio of COVID-19 vaccination when comparing with the reference group.

^b^For the variables having more than two categories, the P-values for overall tests are also provided.

^c^50,000 CNY ≈ 7,370 USD; 150,000 CNY ≈ 22,110 USD; 300,000 CNY ≈ 44,220 USD.

^d^Diseases include epilepsy, malignant tumor, growth retardation, repeated infection, allergic constitution, food allergy, allergic rhinitis, hypothyroidism, chronic urticaria, immune abnormality, cerebral palsy, febrile convulsion, vision abnormality, congenital heart disease, asthma, autism, and other benign diseases.

The results of multivariate analysis showed that children over 4 years [4–5 years: PR = 1.47 (95% CI: 1.33–1.61); 5–6 years: PR = 1.57 (95% CI: 1.43–1.72); 6–7 years: PR = 1.47 (95% CI: 1.32–1.64)], kindergarten pupils (PR = 2.98, 95% CI: 2.19–4.05) or primary school students (PR = 3.18, 95% CI: 2.33–4.35), living in the east (PR = 1.13, 95% CI: 1.07–1.20) or west of China (PR = 1.10, 95% CI: 1.03–1.16), and whose fathers had received at least one dose of a COVID-19 vaccine (PR = 1.69, 95% CI: 1.07–2.67) tended to be vaccinated against COVID-19 after adjusting for other covariates. In contrast, children living in urban areas (PR = 0.94, 95% CI: 0.90–0.98), children with an annual family income < 50,000 CNY (PR = 0.91, 95% CI: 0.84–0.98) or more than 300,000 CNY (PR = 0.90, 95% CI: 0.83–0.97), children who were premature and underwent hospital transfers (PR = 0.85, 95% CI: 0.80–0.91), and children with co-existing diseases (PR = 0.90, 95% CI: 0.83–0.97) were less likely to get vaccinated (Table 2).

**Table 2:** Results of the modified Poisson regression model which evaluated the influential factors of COVID-19 vaccination among preterm-born children aged 3–7 years.

**Variables**	**PR (95% CI)^a^**	***P***
**Age group, years**		
3–4	Reference	
4–5	1.47 (1.33–1.61)	< 0.001
5–6	1.57 (1.43–1.72)	< 0.001
6–7	1.47 (1.32–1.64)	< 0.001
**Education level**		
Pre–kindergarten	Reference	
Kindergarten	2.98 (2.19–4.05)	< 0.001
Primary school	3.18 (2.33–4.35)	< 0.001
**Region**		
Center of China	Reference	
East of China	1.13 (1.07–1.20)	< 0.001
West of China	1.10 (1.03–1.16)	0.002
**Living in urban areas**		
Yes vs. No	0.94 (0.90–0.98)	0.003
**Annual family income, CNY** ^b^		
≤ 50,000	0.91 (0.84–0.98)	0.008
50,000–150,000	Reference	
150,000–300,000	0.97 (0.93–1.02)	0.245
> 300,000	0.90 (0.83–0.97)	0.004
**Natural conception**		
Yes vs. No	1.06 (1.00–1.13)	0.056
**Gestational age, weeks**		
< 32 vs. ≥32	0.96 (0.91–1.01)	0.130
**Referral due to premature delivery**		
Yes vs. No	0.85 (0.80–0.91)	< 0.001
**Atrial septal defect**		
Yes vs. No	0.89 (0.79–1.00)	0.055
**Delay in the first vaccination**		
Yes vs. No	0.98 (0.94–1.02)	0.364
**Currently suffering from diseases** ^c^		
Yes vs. No	0.90 (0.83–0.97)	0.007
**Current weight, kg**	1.00 (1.00–1.01)	0.419
**Being hospitalized in the past year**		
Yes vs. No	1.03 (0.98–1.07)	0.220
**Father's vaccination status**		
Yes vs. No	1.69 (1.07–2.67)	0.024
**Mather's vaccination status**		
Yes vs. No	1.24 (0.96–1.61)	0.098

PR, prevalence ratio; 95% CI, 95% confidence interval; CNY, Chinese yuan renminbi.

^a^Prevalence ratio of COVID-19 vaccination when comparing with the reference group.

^b^50,000 CNY ≈ 7,370 USD; 150,000 CNY ≈ 22,110 USD; 300,000 CNY ≈ 44,220 USD.

^c^Diseases include epilepsy, malignant tumor, growth retardation, repeated infection, allergic constitution, food allergy, allergic rhinitis, hypothyroidism, chronic urticaria, immune abnormality, cerebral palsy, febrile convulsion, vision abnormality, congenital heart disease, asthma, autism, and other benign diseases.

Among the 1,551 children whose parents provided the reasons for COVID-19 vaccination, 1,345 (86.7%) received COVID-19 vaccines following the advice of their kindergartens or primary schools, and 100 (6.4%), 80 (5.2%), and 26 (1.7%) children were vaccinated according to the advice from their communities or doctors, or adopting the suggestion of the public service advertisements. Adverse reactions occurred in 59 (3.8%) of the 1,552 vaccinated children, including fever, injection site pain or induration, chills, fatigue, joint pain, cough, and acute allergic reaction. Among the 59 children with adverse reactions, 43 (72.9%) did not receive special treatment, 14 (23.7%) were only treated with oral medications on the outpatient basis, and only 2 (3.4%) preterm-born children were hospitalized. The decline of the immune system occurred in 3 (5.1%) of the 59 preterm-born children with adverse reactions. Of the 56 fully recovered children, 47 (83.9%) recovered within 7 days (Table 3).

**Table 3:** Information related to COVID-19 vaccination among preterm-born children aged 3–7 years.

**Variables**	***n/N* (%)**
Receipt of COVID-19 vaccines	1,552/1,924 (80.7)
Partially vaccinated	162/1,924 (8.4)
Fully vaccinated	1,390/1,924 (72.2)
**Reasons for getting vaccinated against COVID-19** ^a^	
Kindergarten/school's advice	1,345/1,551 (86.7)
Community's advice	100/1,551 (6.4)
Doctor's advice	80/1,551 (5.2)
Public service advertising	26/1,551 (1.7)
>8 weeks interval between first and second shots	182/1,390 (13.1)
**Reasons for the** >**8 weeks interval between first and**	
**second shots**	
Adverse reactions occurred after the first dose	10/182 (5.5)
Cold	89/182 (48.9)
Other	83/182 (45.6)
Adverse reactions	59/1,552 (3.8)
Fever	22/1,552 (1.4)
Injection site pain	20/1,552 (1.3)
Cold	16/1,552 (1.0)
Injection site induration	5/1,552 (0.3)
Fatigue	5/1,552 (0.3)
Limbs pain	5/1,552 (0.3)
Cough	4/1,552 (0.3)
Acute allergic reaction	3/1,552 (0.2)
**The time with the adverse reaction**	
First dose	33/59 (55.9)
Second dose	20/59 (33.9)
Two doses	6/59 (10.2)
**Dealing with the adverse reaction**	
Not receiving special treatment	43/59 (72.9)
Treated with oral medications	14/59 (23.7)
Hospitalization	2/59 (3.4)
**Outcome after dealing with the adverse reaction**	
Recovery	56/59 (94.9)
The decline of the immune system	3/59 (5.1)
**Recovery duration after having the adverse reaction, days**	
1–7	47/56 (83.9)
8–14	4/56 (7.1)
15–21	2/56 (3.6)
22–30	3/56 (5.4)
**Willingness to vaccinate your child against COVID-19 if the**	
**child did not have relevant contraindications, points** ^b^	
1	63/369 (17.1)
2	61/369 (16.5)
3	87/369 (23.6)
4	57/369 (15.4)
5	101/369 (27.4)

Percentages may not total 100% due to rounding.

^a^Parents of 1,551 preterm-born children provided the reasons for COVID-19 vaccination.

^b^Parents of 369 preterm-born children expressed their willingness to vaccinate their child. The higher the point, the stronger the willingness to vaccinate the child.

**Figure 1:**
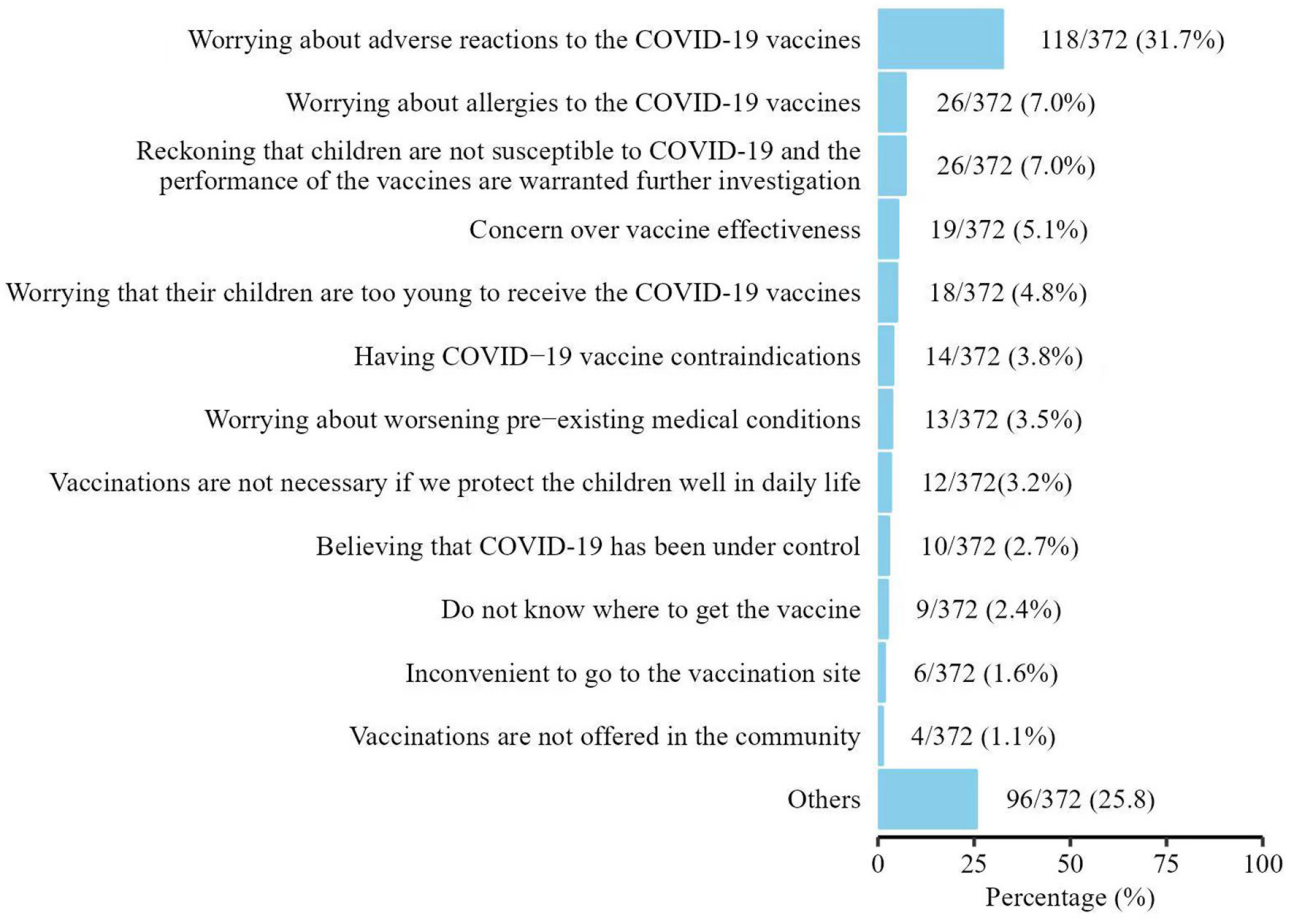
Reasons for not receiving COVID-19 vaccination in preterm-born children in China.

As for reasons of refusing COVID-19 vaccination, 118/372 (31.7%) parents expressed their concerns about the adverse reactions, 26/372 (7.0%) worried about allergies to the COVID-19 vaccines, and 19/372 (5.1%) doubted about the effectiveness of the vaccines. Some parents (26/372; 7.0%) were opinionated that children were not susceptible to COVID-19 infection which needed to be further proved. Some parents reported that they did not know where to get the COVID-19 vaccine (9/372; 2.4%); it was inconvenient for them to get to the vaccination sites (6/372; 1.6%); or vaccines were not available in their communities (4/372; 1.1%). Other reasons such as having COVID-19 contradictions were also mentioned (Figure 1). Among the 369 parents who rated their willingness to vaccinate their children against COVID-19 if their children did not have contraindications, 158 (42.8%) expressed strong willingness, giving 4–5 points (The higher the point is, the stronger the willingness is; Table 3).

## 4. Discussion

With progressive liberation of China's epidemic prevention policy, the COVID-19 pandemic is expected to reach its peak in the next few months. Vaccination is an important way to protect children in this situation. In the study, we estimated the COVID-19 vaccination rate in preterm-born children aged 3–7 in China, and found that 80.7% of the included children had been partially vaccinated against COVID-19 and 72.2% had received two shots. By June 21, 2022, over 98% and 95% of children and adolescents aged 3–17 in China had been partially and fully vaccinated against COVID-19, respectively (17), and the vaccination coverage in children aged 3–11 had reached over 95% in Hainan province and Haidian District of Beijing (18, 19). The vaccination coverage among preterm-born children aged 3–7 can be further improved with more efforts. Regional variations were observed in vaccination coverage, with 75.2, 76.9, and 88.7% children being vaccinated in the west, center, and east of China, respectively. The economic development in the east region is better than that in other regions (20). Consistently, a study in the United States also revealed that the vaccination rate of the influenza vaccine was the highest and increased the most rapidly in the Northeast region, the most economically developed region in the United States (21). The higher vaccination rate in the east of China may be due to the higher population density, better financial security, positive encouragement work, and better COVID-19 vaccination promotion in this region.

Age and education level of children are important factors affecting the vaccination status. We found that the vaccinated children were on average older than the unvaccinated, and 98% of the vaccinated children were kindergarten pupils or primary school students. Teachers supervising, group vaccination, and convenience of receiving vaccines are factors that greatly increased the vaccination rate in school students (22). China has not yet started to vaccinate children under 3 against COVID-19. So if we could vaccinate children under 3 someday in future, it would significantly increase the vaccination rate for those children over 3 who have not yet entered kindergartens.

Our study showed that the COVID-19 vaccination acceptability in children living in urban areas and those who had annual family income < 50,000 CNY was lower than that in children who lived in rural areas and those who had higher annual family income, respectively. A study reported that the COVID-19 trend, public restriction policies and the vaccination status were correlated with economic conditions (23). Living in a high-income household was beneficial to children fully vaccinated (24). Murthy et al. reported that the COVID-19 vaccination rate in some urban areas was lower than that in rural counties in the United States (25). The higher vaccination coverage in rural areas may be due to better COVID-19 vaccination promotion in these areas.

*In vitro* fertilization (IVF) children and some health problems occurring at birth such as some children who underwent hospital transfers and co-existing diseases may all affect the vaccination coverage. The incidences of perinatal mortality and congenital malformation in IVF children are higher than those in average children. The parents of IVF children were more concerned about the health of their children compared with other parents (26, 27). That is also the reason why the COVID-19 vaccination rate in IVF children was relatively lower than that in other children. Referral for premature delivery commonly means delayed administration of the first dose of vaccine, huge cost of hospitalization, longer stay in the hospital, and excessive anxiety about children's health. Delayed administration of the first dose of COVID-19 vaccine usually means that subsequent doses may also be delayed (28). For preterm-born children, hospitalization after discharge from the neonatal intensive care unit and length of stay in the medical institution contribute to the delay of vaccination (29). The co-existence of other diseases reflects the recent health status of children. Caregivers of the children with chronic illness and chronic medication use were reluctant to get their children vaccinated (30). Similar findings were also reported in a survey among adults: respondents with health problems were less likely to get vaccinated compared with those without underlying health problems (31).

We found that parents' vaccination status, especially fathers' vaccination status had an impact on children' vaccination status. The intention to vaccinate children with COVID-19 vaccines aged 0–17 was significantly associated with the parent's/guardian's perception of the COVID-19 vaccine (32). Similarly, the influenza vaccination status of adults in a family had the greatest correlation with children's influenza vaccination status (33). When adults changed from a non-immunized state to an immunized state, their children were more likely to become immunized for influenza (34). Respondents who did not get COVID-19 vaccines were more likely to be highly hesitant, because they either lacked knowledge about the vaccines or were apprehensive about the occurrence of unexpected adverse events (35). Therefore, encouraging parental vaccination is an imperative way to increase the COVID-19 vaccination coverage among children.

Inactivated COVID-19 vaccines are generally safe for preterm-born children. Previous studies reported that adverse reactions of inactivated COVOD-19 vaccines were commonly mild and moderate (8, 36). It was found in our study that only 3.8% vaccinated children had vaccination-related adverse reactions and most of them recovered within 7 days without special treatment. The adverse reactions were mainly mild symptoms, including fever, injection site pain and/or induration, chills, fatigue, joint pain, cough, and acute allergic reaction. That is consistent with the reports of adverse reactions following COVID-19 vaccination among children aged 3–11 in China, which show more than 95% of adverse reactions were mild (37).

Regarding the reasons for not receiving COVID-19 vaccines, some parents held a subjective idea, worrying about the vaccination-related adverse reactions and the effectiveness of the vaccines. The apprehension about the adverse reaction to COVID-19 vaccines was also reported as the main concern of the parents in other studies (38, 39). Meanwhile some parents who lacked knowledge about COVID-19 vaccines worried about the contraindications to the vaccination, leading to parental vaccine hesitancy (40). In such cases, it was particularly important for policy makers, physicians and medical specialists to make a patient and scientific explanation about COVID-19 vaccination to the parents (41). There were only a few objective reasons for vaccine refusal. For instance, only 1.1% of the parents mentioned that COVID-19 vaccines were not accessible or available in their communities. Among the participating parents, 42.8% expressed strong willingness to vaccinate their children, provided their children did not have contraindications. Given gradual liberation of China's COVID-19 prevention policy, we suggest that parents of preterm-born children be provided with common knowledge and professional advice about COVID-19 vaccination through both online and offline ways to encourage them to have their children vaccinated.

This study had some limitations. First, we should have focused on a wider range of ages among preterm-born children due to the heavy workload. Further studies including more age groups of children are necessary. Second, the results of the study would be more representative if more children could have been selected from more hospitals. Finally, the study population mainly included preterm-born Chinese children aged 3–7 and therefore the findings and conclusions may not be generalized to different populations or geographic areas. Multicounty studies are warranted to gain a better understanding of the current status of vaccination coverage among preterm-born children throughout the world.

## 5. Conclusion

In conclusion, COVID-19 vaccination-related adverse reactions rarely occurred and most of them were mild among preterm-born children aged 3–7. The COVID-19 vaccination coverage of these children varies with regions, and the vaccination rate is higher in the east of China. An even higher vaccination coverage in these children can be achieved by more efforts, such as providing more easy-to-understand but professional information about the benefits, contraindications and precautions of COVID-19 vaccination and affording more accessible vaccination sites.

## Data availability statement

The original contributions presented in the study are included in the article/supplementary material, further inquiries can be directed to the corresponding author.

## Ethics statement

This study was approved by ethics committee of the Seventh Medical Center of People's Liberation Army General Hospital. We obtained informed consents from all of the parents of included children to get the necessary information and this survey was completed freely.

## Author contributions

DW conceptualized and designed the study, collected data, interpreted the data, and drafted the manuscript. LL and XM analyzed and interpreted the data and drafted the manuscript. QL and ZF critically conceptualized, designed the study, and revised the manuscript for important intellectual content. YL, LC, A, XP, JS, JY, RJ, ZL, JC, and CL collected data and revised the manuscript. All authors approved the final manuscript as submitted and agree to be accountable for all aspects of the work.
